# GDF11 inhibits adipogenesis of human adipose-derived stromal cells through ALK5/KLF15/β-catenin/PPARγ cascade

**DOI:** 10.1016/j.heliyon.2023.e13088

**Published:** 2023-01-21

**Authors:** Shimin Lin, Lishan Zhong, Jingyi Chen, Zibo Zhao, Rongze Wang, Yexuan Zhu, Junwei Liu, Yanting Wu, Cuifang Ye, Fujun Jin, Zhe Ren

**Affiliations:** a Guangzhou Jinan Biomedicine Research and Development Center, College of Life Science and Technology, Institute of Biomedicine, Jinan University, Guangzhou, China; b School of Biomedical and Pharmaceutical Sciences, Guangdong University of Technology, Guangzhou, China

**Keywords:** GDF11, Adipogenesis, Obesity, KLF15, PPARγ, WNT/β-catenin pathway

## Abstract

Obesity is a metabolic disease characterized by excessive fat storage, and the adipogenic differentiation of adipose-derived stromal cells (ADSCs) is closely linked to its occurrence. Growth differentiation factor 11 (GDF11), a well-known molecule in the field of anti-aging, also has great potential in regulating stem cell differentiation. In this study, we found that GDF11 inhibited adipogenic differentiation of human ADSCs in vitro by activating the WNT/β-catenin and SMAD2/3 pathways while inhibiting the AKT pathway. Moreover, the transcription factor Kruppel-like factor 15 (KLF15) was discovered to be an important downstream factor for GDF11 in inhibiting adipogenesis via the WNT/β-catenin pathway. Furthermore, AlphaFold2 structure prediction and inhibitor-blocking experiments revealed that ALK5 is a functional receptor of GDF11. Collectively, we demonstrated that GDF11 is a potential target for inhibiting adipogenic differentiation and combating obesity.

## 1 Introduction

The imbalance between energy intake and output commonly leads to obesity characterized by excessive fat storage [1,2]. With the improvement in the quality of life, obesity has developed into a global epidemic [3], and the proportion of global obesity increased from 7% in 1980 to 12.5% in 2015, an increase of nearly 80% [4]. Obesity, as a major risk factor for a variety of metabolic disorders, is closely related to insulin resistance, lipid metabolism disorders, liver cirrhosis, coagulopathy, and hypertension [[5], [6], [7]]. In addition, obesity is also involved in the development of certain cancers [[8], [9], [10]]. Therefore, it is particularly important to identify the regulatory factors of adipogenesis.

The signaling pathways regulating adipogenesis mainly include the insulin signaling pathway, glucocorticoid signaling pathway, bone morphogenetic protein signaling pathway, and hedgehog signaling pathway [11,12]. Among many transcription regulators and pathways, peroxisome proliferator-activated receptor γ (PPARγ) and CCAAT enhancer binding proteins alpha (C/EBPα) are the most critical regulators to ensure the adipogenic differentiation of ADSCs and preadipocytes [13,14]. In the early stage of adipogenic differentiation, CCAAT enhancer binding proteins beta (C/EBPβ) and CCAAT enhancer binding proteins delta (C/EBPδ) bind to the C/EBP regulatory elements near the promoter of PPARγ and C/EBPα and activate the expression of PPARγ and C/EBPα [15,16]. PPARγ and C/EBPα, as pluripotent transcription factors, will cascade to activate a large class of adipogenic genes containing C/EBP and PPAR regulatory elements in promoters [17]. The adipogenic differentiation process requires continuous high-level expression of PPARγ and C/EBPα, and their expression depends on the positive feedback regulation between them [16,18].

Growth and differentiation factor 11 (GDF11), a member of the transforming growth factor-beta (TGF-β) superfamily, widely exists in more than 20 tissues, such as skin, heart, skeletal muscle, nervous system, and fat, and plays a key role in human growth and development [19,20]. GDF11 is thought to be highly expressed in the organs and circulation of young individuals, and the expression of GDF11 decreases with the increase of age and the aging process. Increasing the expression level of GDF11 in circulating blood can reverse the aging of muscle and brain [21,22]. GDF11 also plays an important role in the development of the digestive gland, urinary system, and cancer [23,24]. Recently, studies on the relationship between GDF11 and obesity have increased greatly. Administration of recombinant GDF11 protein or plasmid to mice can reduce the weight of the mice and inhibit the formation of white fat [[25], [26], [27]]. It can also improve the hyperglycemia disorder caused by the high-fat diet, relieve the insulin resistance of the mice and maintain the glucose homeostasis [28,29]. GDF11 treatment will reduce mice's food intake and increase energy output [30]. The presence of recombined GDF11 (rGDF11) can reduce the accumulation of lipid droplets and inhibit the expression of fat-related genes [31]. However, another report showed that GDF11 up-regulates the expression of fat mass and obesity-associated protein (FTO), regulates the level of PPARγ methylation to increase the level of PPARγ expression, and promotes the differentiation of bone mesenchymal stem cells (BMSCs) into adipocytes [32]. Thus, the effects of GDF11 on adipogenesis are still controversial.

In this work, we aimed to clarify the effects and regulatory mechanism of GDF11 on adipogenic differentiation and helped to evaluate the translational potential of GDF11 against obesity. The expression of adipogenic-associated genes was significantly down-regulated by GDF11 in human adipose-derived stromal cells (HADSCs). Meanwhile, GDF11 exposure strongly inhibited the formation of lipid droplets in differentiated HADSCs. Through RNA-seq transcriptome analysis, we found that GDF11 treatment inhibited the activation of the AKT pathway during adipogenic differentiation and activated WNT/β-catenin, SMAD2/3, and MAPK signaling pathways. It is worth mentioning that when further analyzing the downstream transcription factors regulated by GDF11, we found that GDF11 inhibited the expression of KLF15, a key regulatory of the WNT/β-catenin pathway and adipogenesis. Furthermore, the KLF15 gene loss-of-function and gain-of-function experiments suggest that KLF15 is a key molecule that links the GDF11-mediated WNT pathway to adipogenesis inhibition. As a result, our research clarifies GDF11's inhibitory effects and deeper regulatory mechanism of action on adipogenesis.

## 2 Materials and methods

### 2.1 Materials

Recombination-GDF11 (rGDF11, Peprotech, Rocky Hill, NJ, USA). IBMX (TargetMol, Boston, USA). Insulin (Macklin Biochemical Co., Ltd., Shanghai, China). Indomethacin, dexamethasone, T3 and Rosiglitazone (Meilun biotechnology Co., Ltd., Dalian, China). RNA extraction reagent (Invitrogen, California, USA). Complete culture medium for human adipose-derived mesenchymal stem cells (Cyagen Biosciences Co., Ltd., Suzhou, China).

### 2.2 Cells and tissues

Human adipose-derived stromal cells (HADSCs) were purchased from Cyagen Biosciences Co., Ltd. (Suzhou, China). The cells were cultured in a complete culture medium (Cyagen Biosciences Co., Ltd., Suzhou, China).

### 2.3 Induction of adipogenic differentiation

Adipogenic differentiation was induced by replacing the growth medium with an adipogenic induction medium after the cells grew to confluence within 24 h. The adipogenic induction medium contained 10% FBS, 0.5 mM IBMX, 125 nM indomethacin, 2 μg/ml dexamethasone, 850 nM insulin, 1 nM T3 and 0.5 μM rosiglitazone. After 3 days of induction, the adipogenic induction medium was replaced by an adipogenic maintenance medium, which was composed of 10% FBS, 850 nM insulin, 1 nM T3 and 0.5 μM rosiglitazone [33]. The maintenance medium should be replaced every two days.

### 2.4 Oil red O staining

The cells were induced to differentiate into adipocytes for eight days. 0.5 g oil red O powder was dissolved in 100 ml isopropanol to make dye, then filtered by filter paper and used within 2 h. Cells were fixed with 4% paraformaldehyde for 15–30 min, then washed with PBS for 3 times. Add oil red O dye and dye in dark for 20 min, and wash with pure water for three times after washing with 60% isopropanol for 5 s.

### 2.5 Lipid fluorescence staining

The cells were induced to differentiate into adipocytes for eight days. The powder of 5 mg BODIPY 493/503 was dissolved in 5 ml anhydrous ethanol, and the storage concentration was 1 mg/ml. In the experiment, the liquid was dissolved in 150 mM NaCl at the ratio of 1:1000 and mixed well. Cells were fixed with 4% paraformaldehyde for 15–30 min, then wash with PBS three times. Add Bodipy dye and dye in dark for 10 min, and then wash with PBS three times.

### 2.6 Cell viability assay

CCK8 assay was used to measure the effect of GDF11 on the proliferation of HADSCs. The HADSCs were cultured with different concentrations of GDF11 for 24 h and 48 h, followed by the CCK8 procedures. The absorbance at 450 nm was recorded by a multimode reader (Bio-Rad, Hercules, CA, USA).

### 2.7 Cell transfection

For siRNA or plasmid transfection, the 5 μl siRNA stock solution (20 μM) or 2 μg plasmid (1 μg/μl) were mixed in 200 μl jetPRIME buffer (PolyPlus), then mixed with 4 μl jetPRIME transfection reagent (PolyPlus). After incubating for 10 min at room temperature, the transfection complex solution was added to the cell culture plate. After 6 h of transfection, the medium was changed to the fresh cell growth medium, and the transfection efficiency was verified by RT-PCR after 48 h incubation.

### 2.8 Transcriptome RNA-seq analysis

HADSCs were cultured in a complete medium for 24 h and then replaced with an adipogenic induction medium containing GDF11 or an adipogenic induction medium without GDF11. The RNA was collected after 72 h of drug treatment, and each well was collected independently. For data processing after sequencing, select |log2Fold Change| greater than 1 and adjust the p-value to less than 0.05 as the threshold to screen for differential genes.

### 2.9 Quantitative real-time PCR (RT-PCR)

RNA extracted from the HADSCs was used to process RNA-to-cDNA transcription for quantitative PCR according to a standard protocol. The amplification was processed for 1 cycle at 95 °C for the 30 s and 40 cycles at 95 °C (5 s) and 60 °C (30 s). The cDNA was amplified by RT-qPCR using specific primer of PPARG sense 5′-TTG TCA CGG AAC ACG TGC A-3′ and anti-sense 5′-GGA GCG GGT GAA GAC TCA TG-3′; CEBPA sense 5′-CCC TCA GCC TTG TTT GTA CTG TAT G-3′ and anti-sense 5′-TTC GTG TTC CTA GGC AAT GCT-3′; FABP4 sense 5′-ACT GGG CCA GGA ATT TGA CGA-3′ and anti-sense 5′-CTC TCG TGG AAG TGA CGC CT-3′; PLIN1 sense 5′-GTC AGC CGG AGT GAG TGT TG-3′ and anti-sense 5′-GGT GAG GCC TTT GTT GAC TGC-3′; LPL sense 5′-AGC TAT CCG CGT GAT TGC A-3′ and anti-sense 5′-ACT AGC TGG TCC ACA TCT CCA AG-3′; ADIPOQ sense 5′-TGC AGT CTG TGG TTC TGA TTC CA-3′ and anti-sense 5′-GTC ATG ACC GGG CAG AGC TA-3′; STAT5 sense 5′-TGT CTC TGA GGT CAC AAA ACC TG-3′ and anti-sense 5′-TCA GCT GCC TGC GAT AGA CC-3′; ZFP36L1 sense 5′-GGA CAA CTC AAG ACG CCT GC-3′ and anti-sense 5′-CTG GGG GAA AGG GGT TGA GC-3′; KLF7 sense 5′-TCC CCT TTT CCT GGC AGT CG-3′ and anti-sense 5′-GAA ACC CTC CCC CGA ACA CA-3′; KLF15 sense 5′-GGT GAA AAG CGT CCC CCA CT-3′ and anti-sense 5′-TGT CTG GGA AAC CGG AGG AG-3′; CEBPB sense 5′-CGC TTA AAG ATG TTC CTA CGG GC-3′ and anti-sense 5′-CCC CAA AAG GCT TTG TAA CCA TTC T-3′; CEBPD sense 5′-AGC TGT CGG CTG AGA ACG AG-3′ and anti-sense 5′-GTT ACC GGC AGT CTG CTG TC-3′; ACTR2B sense 5′-CTC CTC TGG GGA TCG CTG T-3′ and anti-sense 5′-CTC CCA GTT GGC GTT GTA GT-3′; ACTR2A sense 5′-CCT CGG ACT TTA GGT GTC TGG G-3′ and anti-sense 5′-TGC AGC AGC TCC CAT TTT CC-3′; ALK4 sense 5′-GAG CAC GGG TCC CTG TTT GA-3′ and anti-sense 5′-TCG CAT CAT CTT CCC CAT CAC-3′; ALK5 sense 5′-GGG GCG ACG GCG TTA CAG TGT TTC TGC CAC-3′ and anti-sense 5′-TGA GAT GCA GAC GAA GCA CAC TGG TCC AGC-3′; ALK7 sense 5′-CGC ACT TCA AAA GGG TGT CG-3′ and anti-sense 5′-GAT GCC CAA CAT GCT CCT T-3′.

### 2.10 Western blot

Proteins in cells were extracted using RIPA, and the concentration was measured by a BCA protein assay kit (Beyotime, Shanghai, China). The samples were then processed by SDS-PAGE and electrophoretically transferred onto a polyvinylidene fluoride membrane that subsequently was blocked with 5% BSA solution for 1 h and then treated with the intended primary antibodies (1:1000) at 4 °C overnight. On the second day, the membrane was incubated with an HRP-labeled secondary antibody (1:5000) for 1 h. The membrane was subjected to a ChemiDoc MPTM Imaging System (Bio-Rad, Hercules, CA, USA). The following antibodies were used: anti-β-actin antibody (Cell Signaling Technology, 4970), anti-PERILIPIN antibody (Cell Signaling Technology, 9349), anti-PPARγ antibody (Cell Signaling Technology, 2435), anti-FABP4 antibody (Cell Signaling Technology, 2120), anti-CEBPα antibody (Cell Signaling Technology, 2843), anti-pAKT antibody (Cell Signaling Technology, 4060), anti-AKT antibody (Cell Signaling Technology, 9272), anti-pERK1/2 antibody (Cell Signaling Technology, 4370), anti-ERK1/2 antibody (Cell Signaling Technology, 4695), anti-pP38 antibody (Cell Signaling Technology, 4511), anti-P38 antibody (Cell Signaling Technology, 9212), anti-pJNK antibody (Cell Signaling Technology, 9255), anti-JNK antibody (Cell Signaling Technology, 9252), anti-pSMAD1/5 antibody (Cell Signaling Technology, 9516), anti-pSMAD2 antibody (Cell Signaling Technology, 18338), anti-pSMAD3 antibody (Cell Signaling Technology, 9520), anti-SMAD2/3 antibody (Cell Signaling Technology, 8685), anti-rabbit IgG, HRP-linked Antibody (Cell Signaling Technology, 7074) and anti-mouse IgG, HRP-linked Antibody (Cell Signaling Technology, 7076).

### 2.11 AlphaFold2 and molecular dynamics simulation

AlphaFold2 was run on Google's jupyter Notebook, using the publicly available source code of AlphaFold2 [34]. Protein dynamics simulations were performed using Desmond by first subjecting the protein to conventional processing and then using OPLS4 force field minimization to remove atomic collisions. The SPC force field within an Orthorhombic box of size 10 × 10 × 10 Å was added and counterions (Cl- and Na+) with a density of 0.15 mol were added to simulate the in vivo environment. Finally, the MD simulation was run for 100 ns at a 300 K temperature and standard pressure (1.01325 bar), with 10 ps as the recording interval.

### 2.12 Statistical analysis

Unpaired Student's t-test (two-tailed) was used to determine the statistical differences between the two groups. One-way ANOVA with Tukey's multiple comparisons test was used to determine the statistical differences between multiple groups. Data are presented as mean ± standard deviation (SD). n value was 3 if it was not specified. Statistical differences were defined as *P < 0.05, **P < 0.01, ***P < 0.001, and ****P < 0.0011, n.s, not significant.

## 3 Result

### 3.1 GDF11 inhibits adipogenic differentiation of HADSCs

**Fig. 1:**
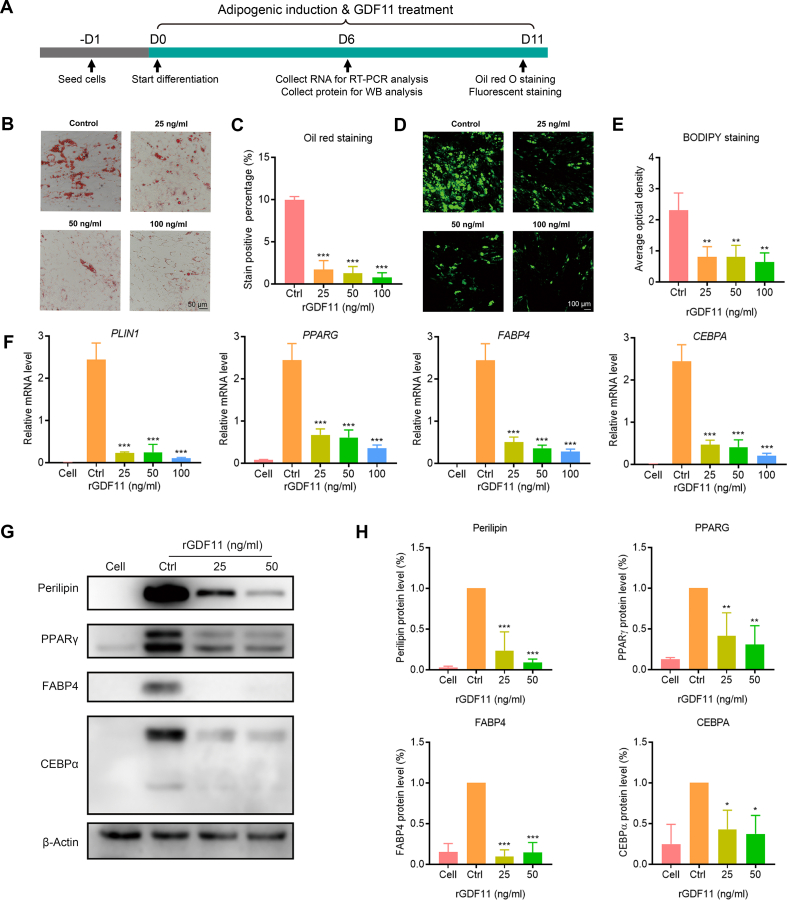
Inhibitory effects of GDF11 on adipogenic differentiation. (A) Cell administration and sample collection pattern. (B) The formation of lipid droplets was detected by oil red O staining. (C) Statistical analysis of the oil red O positive-stain percentage. (D) BODIPY fluorescence staining was used to detect the formation of neutral lipids. (E) Statistical analysis of the average optical density (AOD) of BODIPY fluorescence signal. (F) The mRNA levels of adipogenic-specific genes after GDF11 treatment were analyzed by RT-qPCR. (G) The expression levels of adipogenesis-associated protein Perilipin, PPARγ, FABP4, and CEBPα after GDF11 treatment. Uncropped images were provided in the supplementary file. (H) Statistical analysis of Perilipin, PPARγ、FABP4, and CEBPα levels. Data are expressed as mean ± SD (n = 3). *P < 0.05, **P < 0.01, ***P < 0.001. (For interpretation of the references to colour in this figure legend, the reader is referred to the Web version of this article.)

Firstly, we evaluated the effects of GDF11 on adipogenic differentiation in an in vitro adipogenesis model of HADSCs (Fig. 1A). The formation of lipid droplets was measured by oil red O staining, and the staining area of lipid droplets in the GDF11 treated group was only one-fifth of that in the control group, indicating that the adipogenic differentiation was strongly inhibited by GDF11 (Fig. 1B and C). The result of neutral lipids fluorescence staining further indicated that GDF11 exposure could inhibit the adipogenesis of the HADSCs (Fig. 1D and E). Meanwhile, the mRNA levels of the adipogenic specific genes, including PPARG, CEBPA, Fatty acid binding protein 4 (FABP4), and Perilipin 1 (PLIN1) in HADSCs were significantly down-regulated by GDF11 exposure in a dose-dependent manner (Fig. 1F). The down-regulation of the adipogenic-specific genes was further confirmed by the Western blot assay. The protein levels of Perilipin, PPARγ、CEBPα, and FABP4 were significantly decreased in HADSCs after GDF11 treatment (Fig. 1G and H). Furthermore, we investigated the effects of lower-dose GDF11 on HADSCs adipogenic differentiation. The oil red O staining and RT-PCR assay results consistently demonstrated that the lowest effective concentration of GDF11 on adipogenic differentiation was as low as 3.13 ng/ml (Supplementary Fig. S1). Taken together, these results suggest that GDF11 was a potent inhibitor of adipogenic differentiation.

### 3.2 GDF11 regulates the expression of adipogenic transcription factors

**Fig. 2:**
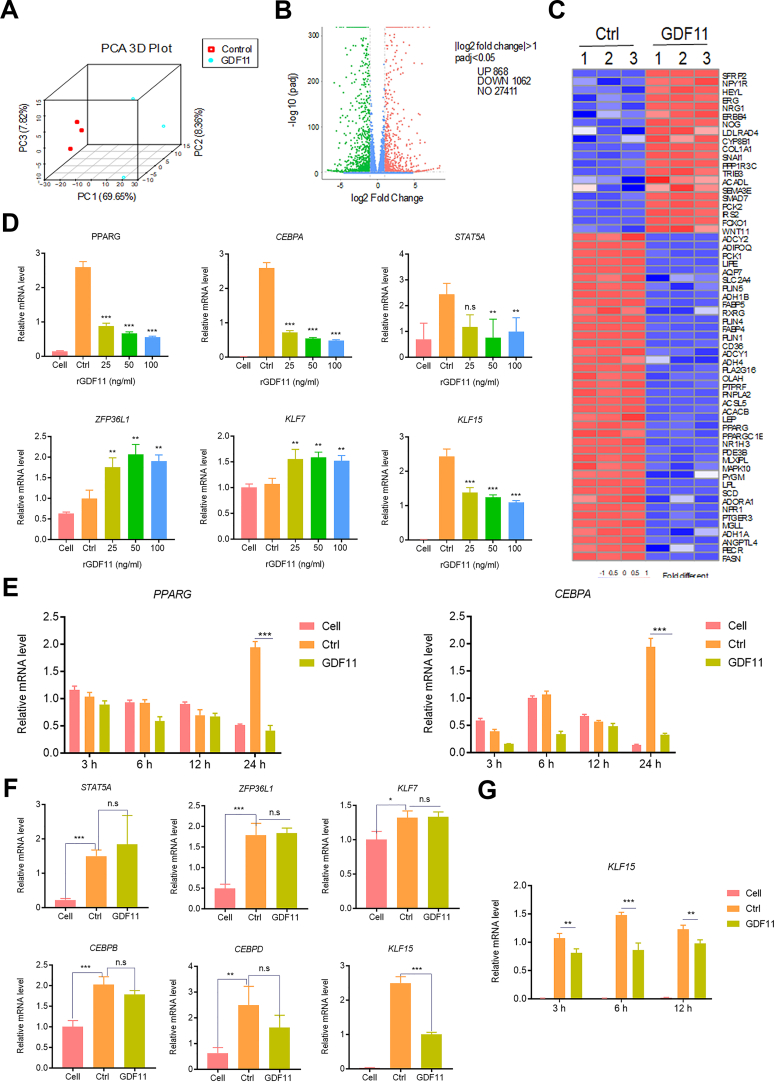
RNA-seq analysis after GDF11 treatment and RT-qPCR verification of key transcription factors. (A) The repeatability of samples was analyzed by PCA. (B) The different gene groups after GDF11 (25 ng/ml) treatment showed by the volcano map. (C) Thermography of differential expression of adipogenic genes under GDF11 (25 ng/ml) treatment. (D) The mRNA levels of adipogenic transcription factors at 72 h by RT-qPCR. (E) The mRNA expression levels of PPARG and CEBPA at different time points. (F) Expression of transcription factors in HADSCs treated with GDF11 (25 ng/ml) and for 24 h. (G) The expression levels of KLF15 at different time points. Data are expressed as mean ± SD (n = 3). **P < 0.01, ***P < 0.001; ns, no significance.

Next, to explore the molecular mechanism of GDF11 inhibiting adipogenic differentiation, high-throughput RNA sequencing (RNA-seq) of GDF11-treated HADSCs was performed. Principal component analysis (PCA) analysis showed that there was good repeatability in the test sample group, which enhanced the reliability of subsequent RNA-seq statistical analysis (Fig. 2A). A total of 1930 genes with log2 (fold change) > 1 were identified, including 868 upregulated and 1062 downregulated genes (Fig. 2B). The differential genes contained a large number of adipogenic-related genes, among which the heat map lists 60 adipogenic-associated genes involved in the PPARγ signaling pathway, fatty acid metabolism, and insulin resistance (Fig. 2C). The RT-qPCR assay was carried out at 72 h after GDF11 treatment. The most important transcription factors of adipogenic differentiation and a large number of adipogenic-specific genes were expressed (Fig. 2D). To find out whether these transcription factors are really involved in the inhibition of PPARγ by GDF11 and whether they play an important role in the connection between GDF11 and PPARγ, the time of GDF11 treatment was shortened and the expression levels of these transcription factors before PPARγ was inhibited by GDF11 were detected by RT-qPCR. The mRNA expression levels of PPARG and CEBPA were significantly inhibited by GDF11 (25 ng/ml) at 24 h (Fig. 2E). The results showed that except for KLF15, the gene expression levels of signal transducer and activator of transcription 5A (STAT5A), ZFP36 Ring Finger Protein-Like 1 (ZFP36L1), Kruppel-like factor 7 (KLF7), CEBPB, and CEBPD remained unchanged after 24 h of GDF11 (25 ng/ml) treatment (Fig. 2F). The adipogenesis-positive regulator KLF15 was significantly down-regulated at 3 h, 6 h, 12 h, and 24 h after treatment with GDF11 (25 ng/ml), with the best effect at 24 h (Fig. 2G). These results indicate that KLF15 can be further explored as downstream transcription factors of GDF11 inhibiting adipogenesis.

### 3.3 GDF11 inhibited the activation of the AKT pathway

**Fig. 3:**
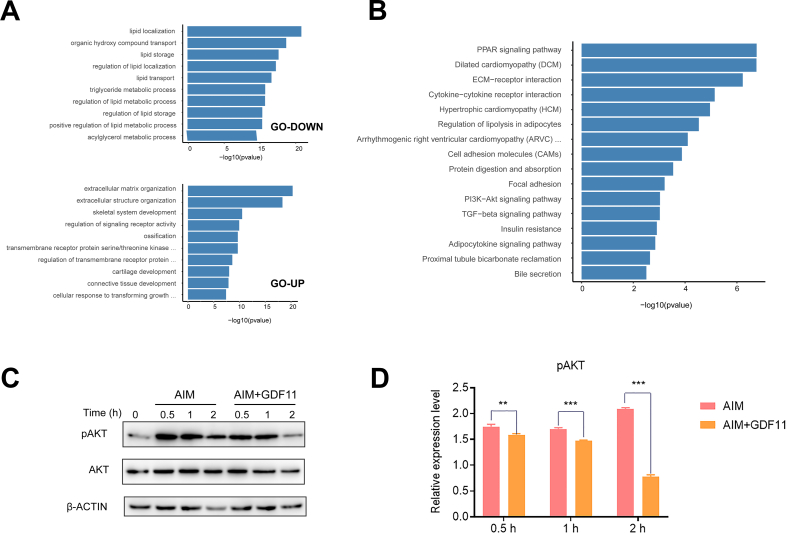
GDF11 inhibited the activation of the AKT pathway. (A) GO cluster analysis of differential genes. (B) KEGG enrichment analysis of differentially expressed genes. (C) The down-regulation of pAKT in HADSCs after GDF11 (25 ng/ml) treatment. Uncropped images were provided in the supplementary file. (D) Statistical analysis of pAKT levels. Data are expressed as mean ± SD (n = 3). **P < 0.01, ***P < 0.001.

The enrichment of differential genes can further explore the effect and target of GDF11 on adipogenesis. In the Gene Ontology (GO) analysis, the top ten most significant clusters were almost all related to fat metabolism, and they were involved in the location, storage, transportation, and metabolism of fat (Fig. 3A). In the Kyoto Encyclopedia of Genes and Genomes (KEGG) cluster analysis, PPARγ, the most important signaling pathway of adipogenesis, was first enriched. Secondly, the enriched signaling pathways included PI3K/AKT pathway, TGF-β signaling pathway, insulin resistance pathway, and adipocytokine signaling pathway (Fig. 3B). PI3K/AKT is a component of the insulin signaling pathway, and activating the AKT pathway will promote the adipogenic differentiation of adipose stem cells [35]. We find that GDF11 (25 ng/ml) exposure remarkably suppressed the level of the phosphorylated AKT at 0.5 h, 1 h, and 2 h. Indicating that GDF11 could inhibit the activation of the AKT pathway (Fig. 3C and D).

### 3.4 GDF11 inhibited the ERK1/2 pathway but activated the p38 signal pathway

**Fig. 4:**
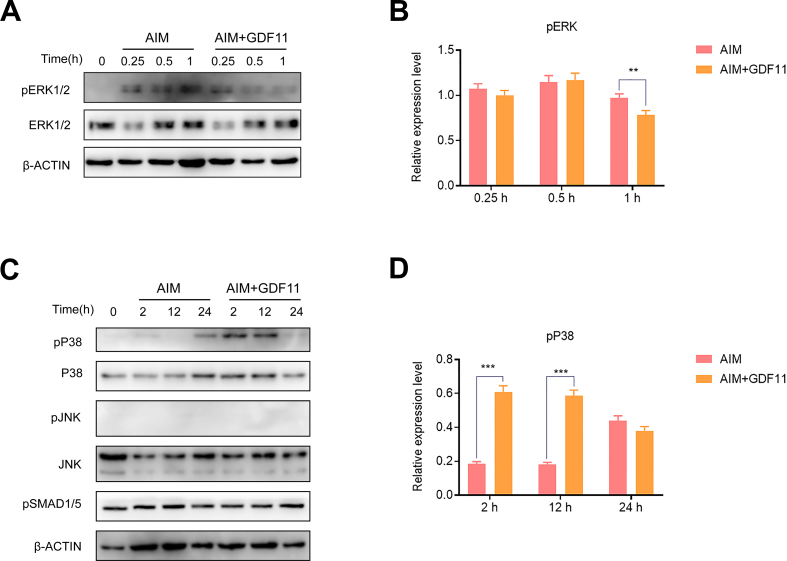
Regulation of MAPK signal pathway and SMAD1/5 signal pathway by GDF11. (A) The inhibition of pERK1/2 after GDF11 (25 ng/ml) treatment. Uncropped images were provided in the supplementary file. (B) Statistical analysis of pERK levels. (C) p38 was up-regulated after GDF11 (25 ng/ml) treatment. Uncropped images were provided in the supplementary file. (D) Statistical analysis of p-p38 levels. Data are expressed as mean ± SD (n = 3). **P < 0.01, ***P < 0.001.

GDF11 binds to its receptors and activates SMAD and non-SMAD signaling pathways, representing TGF-β typical and atypical signaling pathways downstream of superfamily members. It has been reported that GDF11 can activate SMAD1/5/8 [36,37]. In addition to the SMAD proteins, TGF-β superfamily members also activate other non-SMAD signals, such as MAP kinases (ERK, p38, and JNK) [38]. Western blot was used to explore the regulation of GDF11 on ERK1/2, P38, JNK, and SMAD1/5 pathways. The result showed that GDF11 inhibited the ERK1/2 pathway (Fig. 4A and B). However, GDF11 activated p38 and had no effect on the SMAD1/5 signal pathway (Fig. 4C and D).

### 3.5 GDF11 activated SMAD2/3 signaling pathway

**Fig. 5:**
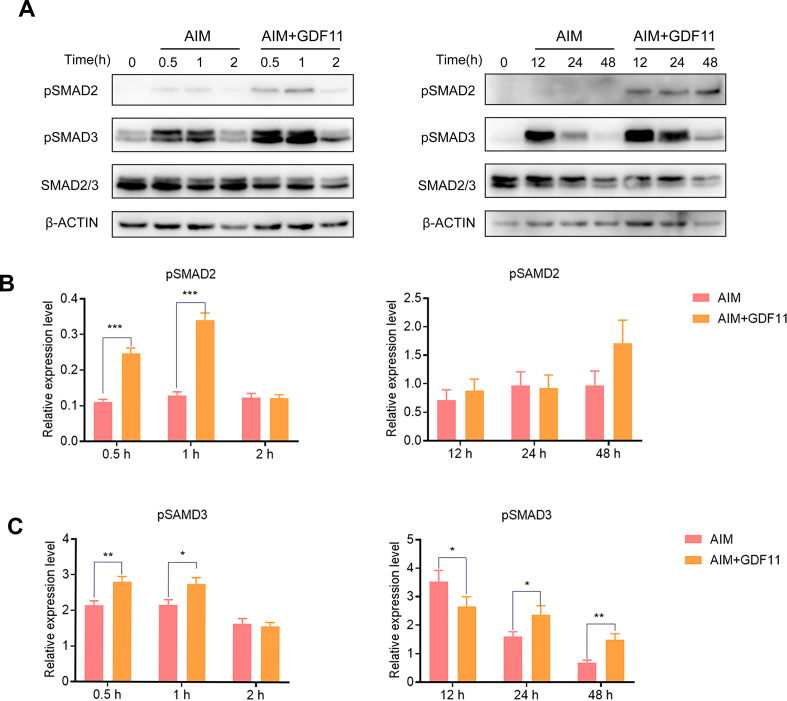
GDF11 activated SMAD2/3 signaling pathway. (A) The effect of GDF11 GDF11 (25 ng/ml) exposure on the SMAD2/3 pathway showed by the Western blot. Uncropped images were provided in the supplementary file. (B) Statistical analysis of pSMAD2 levels. (C) Statistical analysis of pSMAD3 levels. Data are expressed as mean ± SD (n = 3). *P < 0.05, **P < 0.01, ***P < 0.001.

The pathway enriched by KEGG also includes the TGF-β pathway. While GDF11 acts as a TGF-β family member, TGF-β/SMAD is its classical signal pathway. A recent study has shown that human bone marrow-derived mesenchymal stem cells (hMSCs) and 3T3-L1 pre-adipocytes treated with GDF11 can activate the TGF-β/SMAD2/3 signaling pathway [39]. In contrast to the AKT pathway, SMAD 2/3 pathway was activated after GDF11 treatment, as showed by the up-regulation of the pSMAD2 and pSMAD3 at 0.5 h, 1 h and 2 h, and the activation of SMAD2/3 was still significant when GDF11 was treated for 12 h, 24 h and 48 h (Fig. 5A–D).

### 3.6 GDF11 regulates the WNT/β-catenin pathway via KLF15

**Fig. 6:**
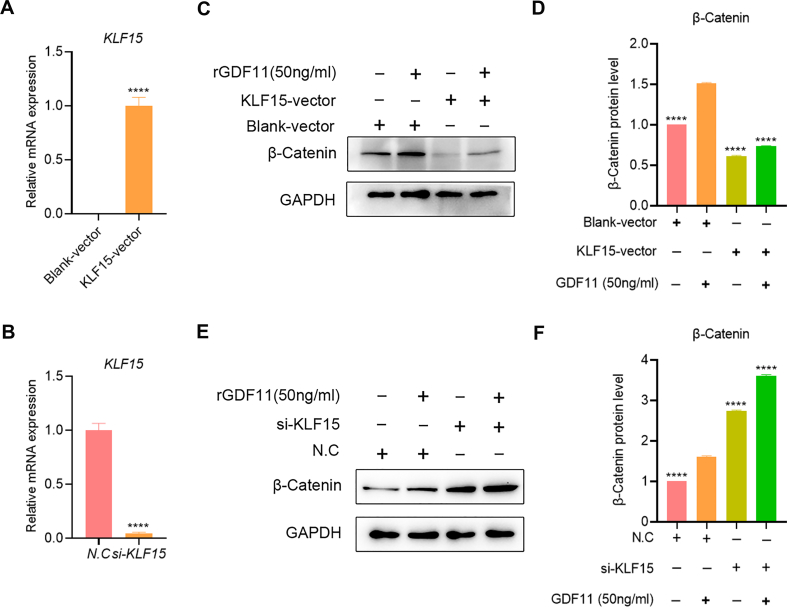
GDF11 regulates the WNT/β-catenin pathway via KLF15. RT-PCR analysis of the KLF15 gene overexpression (A) or knockdown (B) efficiency. (C) The effect of GDF11 (25 ng/ml) exposure on the WNT/β-catenin pathway in blank vector or KLF15 overexpression vector transfected HADSCs was evaluated by Western blot. Uncropped images were provided in the supplementary file. (D) Statistical analysis of β-catenin protein levels. (E) The effect of GDF11 (25 ng/ml) exposure on the WNT/β-catenin pathway in scramble siRNA or KLF15 siRNA transfected HADSCs was evaluated by Western blot. Uncropped images were provided in the supplementary file. (F) Statistical analysis of β-catenin protein levels. Data are expressed as mean ± SD (n = 3). ****P < 0.0001.

WNT/β-catenin pathway, whose activation blocks adipogenesis, has been reported to involve in GDF11-mediated adipogenesis inhibition^[29]^. Meanwhile, KLF15 has been reported to block the WNT/β-catenin pathway [40]. Therefore, we explored whether the activation of the WNT/β-catenin pathway by GDF11 is regulated by KLF15. The results showed that GDF11 treatment could activate the WNT/β-catenin pathway in HADSCs, as indicated by the significantly increased β-catenin protein expression levels (Fig. 6). Next, we used plasmid to mediate the overexpression of the KLF15 gene (Fig. 6A) and siRNA to silence the expression of the KLF15 gene (Fig. 6B) in HADSCs to explore the effects of the loss-of-function or gain-of-function of KLF15 gene on WNT/β-catenin signaling pathway mediated by GDF11. Interestingly, the results showed that overexpression of GDF11 completely reversed the up-regulation of β-catenin mediated by GDF11 (Fig. 6C and D). Moreover, the knockdown of KLF15 could further induce the up-regulation of β-catenin mediated by GDF11 (Fig. 6E and F). Therefore, these results indicate that KLF15 is an important molecule that links the GDF11-mediated WNT/β-catenin pathway activation.

### 3.7 Inhibition of ALK5 receptor relieved the activity of GDF11

**Fig. 7:**
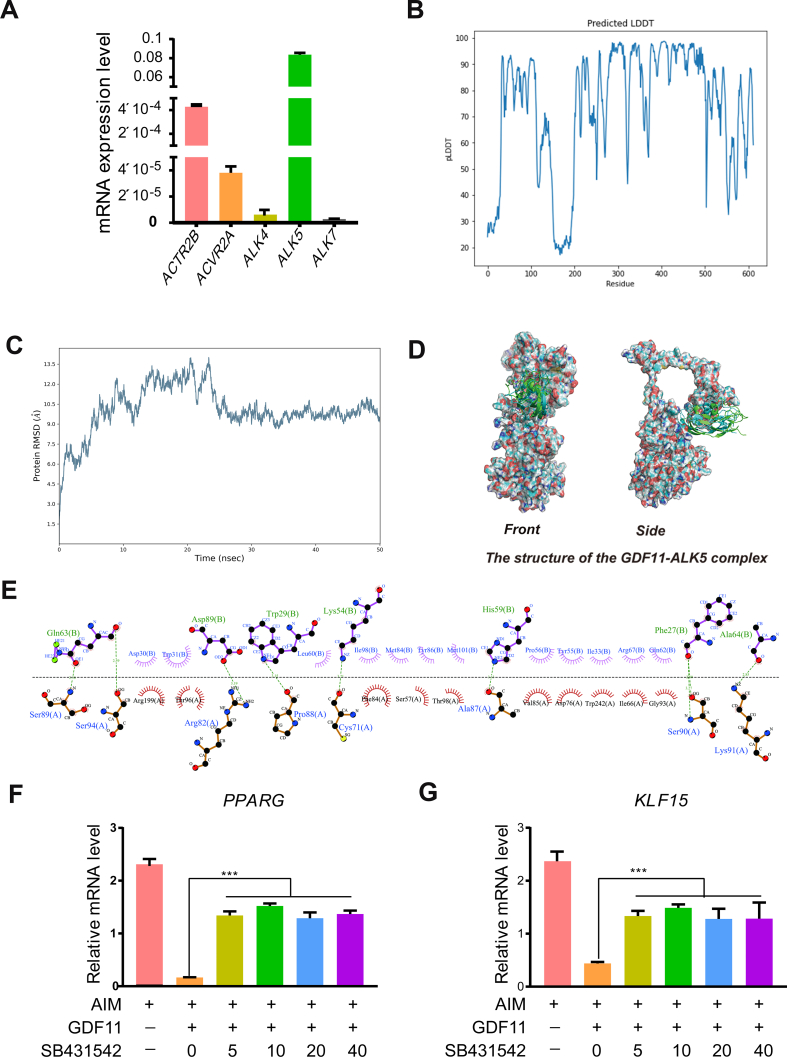
ALK5 was a GDF11 receptor in HADSCs. (A) The expression of GDF11 receptor on HADSCs cell membrane. (B) Confidence scores of ALK5-GDF11 complex for prediction by amino acid order. (C) Root Mean Square Deviation (RMSD) of ALK5-GDF11 complex for 50 ns. (D) The 3D structure of ALK5-GDF11 complex. The green line indicates GDF11 in the initial conformation, and the blue line indicates GDF11 in the stabilized conformation. (E) Post-2D amino acid binding plot of GDF11 with ALK5. Chain A stands for ALK5, and Chain B stands for GDF11. The mRNA levels of PPARγ (F) and KLF15 (G) in adipogenic medium induced HADSCs treated with ALK4/5 inhibitor 50 nM SB-431542 and 100 ng/ml GDF11 for 3 days. Data are expressed as mean ± SD (n = 3). ***P < 0.001. (For interpretation of the references to colour in this figure legend, the reader is referred to the Web version of this article.)

GDF11 is a transmembrane heterodimer receptor composed of activin type I receptors (ALK4, ALK5, ALK7) and activin type II receptors (ACTRIIA, ACTRIIB) [34]. Among the type I receptors of HADSCs, the expression of ALK5 was the highest and ALK7 the lowest (Fig. 7A). The structure of the GDF11-ALK5 complex was predicted using AlphaFold2 [34], with confidence scores plotted in amino acid order in Fig. 7B. Except for the 150–200 amino acid region with the lowest confidence score, the overall model prediction reliability is high, indicating that the structure is reliable. 50 ns dynamics were performed using the predicted results of AlphaFold2 as the initial conformation. The extent to which the GDF11-ALK5 complex protein deviates from the mean and the stability of the ensemble were assessed from Root Mean Square Deviation (RMSD [34]. The RMSD map of the protein backbone was drawn using Ca atoms (Fig. 7C). In the early stage of dynamics, there is a large fluctuation. After 25 ns, the fluctuation is small and maintained at about 10 Å, indicating that the binding system has reached a new steady state and the binding is stable. It was found from the initial conformation (Fig. 7D and E) that the active amino acids of GDF11 form hydrogen-bonding interactions or salt bridges with Asp81, Arg82, Pro88, Ser89, Ser90, and Lys91 on ALK5. It is worth noting that these amino acid sites are also located precisely in the extracellular region of ALK5. Taking the dynamics last frame conformation as the post-stabilized conformation, the spatial position of GDF11 binding to ALK5 did not change much compared with the initial conformation. The active amino acids of GDF11 are still tightly bound to the amino acids in the extracellular domain of ALK5, forming a stable interaction force. Therefore, the extracellular domain of ALK5 may be the main binding domain for GDF11. Next, to explore whether the GDF11's activity depended on the binding with ALK5, a selective inhibitor of ALK5, SB-431542, was used to block the functions of ALK5. The HADSCs were treated with 50 nM SB-431542 and 100 ng/ml GDF11 in an adipogenic differentiation induction medium for 3 days. The RT-qPCR results showed that the addition of SB-431542 could relieve the inhibitory effect of GDF11 on PPARγ and KLF15 (Fig. 7F and G). These results preliminarily showed that ALK5 was a GDF11 receptor that medicated the adipogenic inhibition activity of GDF11.

## 4 Discussion

In this study, we used the cocktail method to induce adipogenic differentiation of HADSCs in vitro and in a mixed system of various components required for adipogenic differentiation [[41], [42], [43]]. The HADSCs used in this study are derived from human adipose tissue and belong to primary cells, which is a representative model for adipogenic differentiation. The phenotype verification and mechanism exploration of HADSCs can eliminate the species differences under the effect of GDF11.

There has some evidence that GDF11 played a role in adipogenesis. GDF11 can alleviate a series of symptoms caused by a high-fat diet, such as weight gain, hyperglycemia disorder, insulin resistance, and increased white fat production [28,29,44]. In mechanism, pSMAD2, pAKT, pFOXO1, and pAMPK were activated in the white fat of GDF11 overexpressed mice [28]. In the existing reports, the adipogenic effect caused by GDF11 in vitro cell model is controversial. GDF11 reduced the mRNA levels of PPARG and CEBPA in human mesenchymal stem cells, and activated the classical TGF-β/SMAD2/3 pathway in 3T3-L1 cells [31]. Another report has given the different adipogenic inhibitory mechanisms of GDF11. GDF11 did not affect the total protein level of PPARγ but increased PPARγ SUMOylation, which inhibited adipogenic differentiation and promoted osteogenic differentiation of BMSC. However, GDF11 promoted adipogenic differentiation in some studies. GDF11 regulated the level of PPARγ methylation by upregulating the expression of FTO to improve the expression level of PPARγ and promote adipogenic differentiation [32]. Although the inhibitory effect of GDF11 on adipogenesis in vitro is controversial, it may be caused by the use of cells from different sources as models in the experiment. It is important that the key evidence of GDF11 inhibiting the adipogenic effect is shown in this paper. High-throughput RNA sequencing (RNA-seq) of HADSCs and GDF11-treated HADSCs was performed. The differential genes contained a large number of adipogenic-related genes involved in the PPARγ signaling pathway, fatty acid metabolism, and insulin resistance. In addition, the adipogenic inhibitory effect of GDF11 was also verified by RT-qPCR. GDF11 is convincing as an adipogenic inhibitor.

**Fig. 8:**
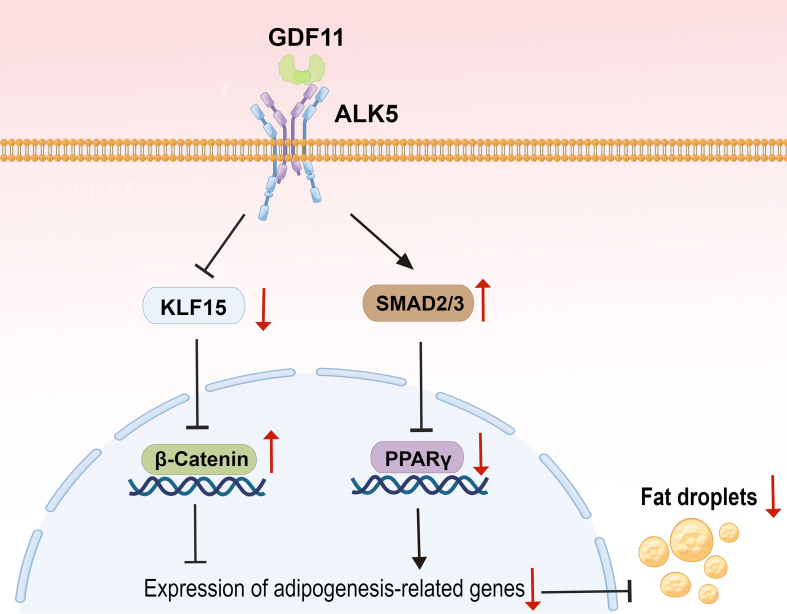
Schematic diagram of GDF11 inhibiting adipogenic differentiation.

KLF15 is an important adipogenic factor. The expression of KLF15 can be detected when MSCs and MEFs cells differentiate into adipocytes for 12 h and reach a peak on D2 of differentiation [45]. KLF15 deficient mice lost weight, reduced fat weight, and increased glucose tolerance [46]. KLF15-deficient mice could slow down the absorption of lipids or other nutrients and fight against insulin resistance induced by a high-fat diet [47]. KLF15 regulates the expression of important genes for adipogenic differentiation and inhibits lipolysis, and specifically, knocking out KLF15 in adipocytes would reduce fat deposition [48]. Besides, KLF15 knockdown in MEFs inhibited adipogenic differentiation [49]. KLF15 is the downstream target of C/EBPβ and C/EBPδ. KLF15 could activate the GLUT4 promoter and regulate the expression of acetyl-CoA synthase, thus acting on adipogenesis [50]. KLF15 also promoted the expression of another adipogenic forward transcription factor KLF3 [51]. Moreover, the WNT/β-catenin pathway, whose activation blocks adipogenesis, has been reported to block by KLF15 [40]. In this study, KLF15 was found to be an important molecule that links the GDF11-mediated WNT/β-catenin pathway activation. Meanwhile, we find that the AKT pathway was inhibited under the treatment of GDF11. Importantly, PI3K/AKT activated by the insulin signaling pathway could promote the expression of KLF15 [52]. Therefore, GDF11 may act on KLF15 by inhibiting the AKT pathway. Furthermore, we identified ALK5 as an important receptor of GDF11 in HADSCs by both AlphaFold2 structure modeling and receptor inhibition experiments. When the GDF11 receptor ALK5 was blocked by SB-431542, it relieved the inhibition of PPARG and KLF15 by GDF11. Therefore, our study primarily clarified the regulatory mechanism of GDF11 on adipogenesis and demonstrated that the cascade reaction ALK5/KLF15/β-catenin axis might be an important part of GDF11's inhibitory effect on adipogenesis (Fig. 8).

## 5 Conclusions

Our study clarified the effects and regulatory mechanisms of GDF11 on adipogenic differentiation and demonstrated that GDF11 is a potential target for inhibiting adipogenic differentiation and combating obesity.

## Author contribution statement

Shimin Lin: Performed the experiments; Wrote the paper.

Lishan Zhong, Jingyi Chen: Performed the experiments; Analyzed and interpreted the data; Wrote the paper.

Zibo Zhao, Rongze Wang, Junwei Liu: Performed the experiments.

Yexuan Zhu, Yanting Wu: Analyzed and interpreted the data.

Cuifang Ye, Zhe Ren: Conceived and designed the experiments.

Fujun Jin: Conceived and designed the experiments; Wrote the paper.

## Funding statement

Zhe Ren Zhe Ren was supported by This research was funded by the 10.13039/501100001809National Natural Science Foundation of China [81872908], the 10.13039/501100003453Natural Science Foundation of Guangdong Province [2019A1515010046].

## Data availability statement

Data will be made available on request.

## Declaration of interest's statement

The authors declare that they have no known competing financial interests or personal relationships that could have appeared to influence the work reported in this paper.
