# Supplementary material for: GDF11 inhibits adipogenesis of human adipose-derived stromal cells through ALK5/KLF15/β-catenin/PPARγ cascade
Source: Heliyon. 2023 Jan 21;9(2):e13088. doi: 10.1016/j.heliyon.2023.e13088 (PMC9900277; doi:10.1016/j.heliyon.2023.e13088)

Supplementary Information

GDF11 inhibits adipogenesis of human adipose-derived stromal cells through ALK5/KLF15/β-catenin/PPARγ cascade

Shimin Lin, Lishan Zhong, Jingyi Chen, Zibo Zhao, Rongze Wang, Yexuan Zhu, Junwei Liu, Yanting Wu, Cuifang Ye*, Fujun Jin*, Zhe Ren*

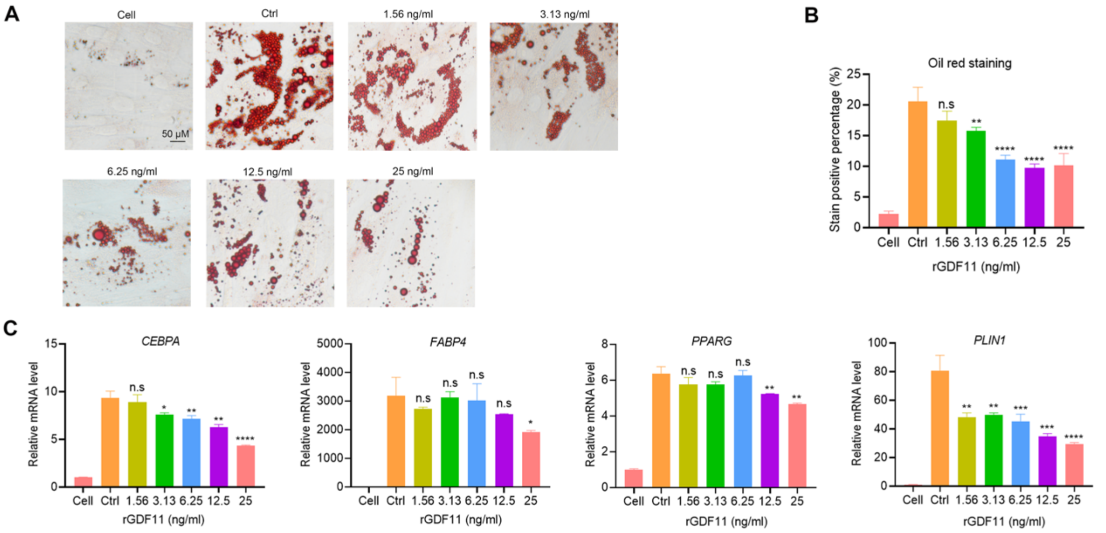

Supplementary Figure 1. The minimum effective doses of GDF11 on adipogenic differentiation. (A) The formation of lipid droplets was detected by oil red O staining. (B) Statistical analysis of the oil red O positive-stain percentage. (C) The mRNA levels of adipogenic-specific genes after GDF11 treatment were analyzed by RT-qPCR. Data are expressed as mean ± SD (n=3). n.s, not significant, *P < 0.05, **P < 0.01, ***P < 0.001, ****P < 0.001.

Uncropped images for Figure 1G

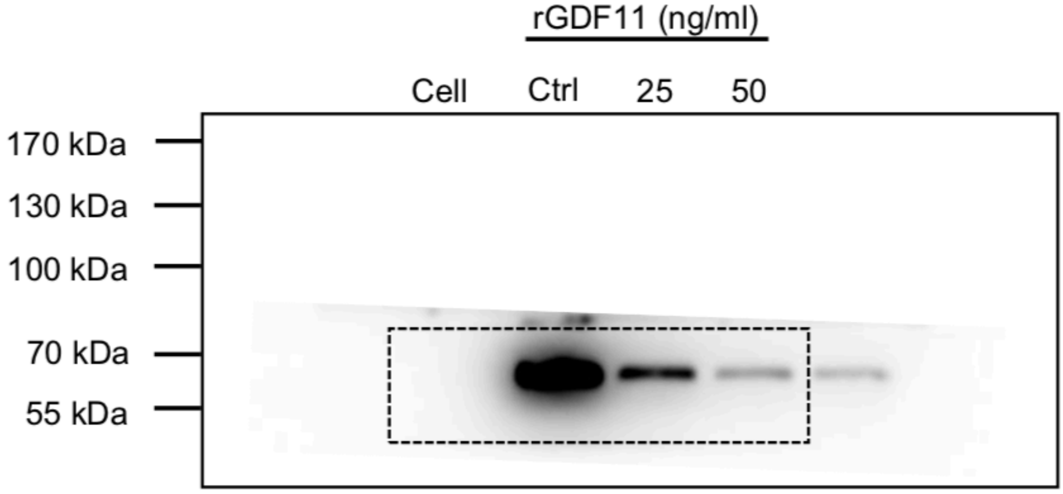

Anti-Perilipin (62kDa)

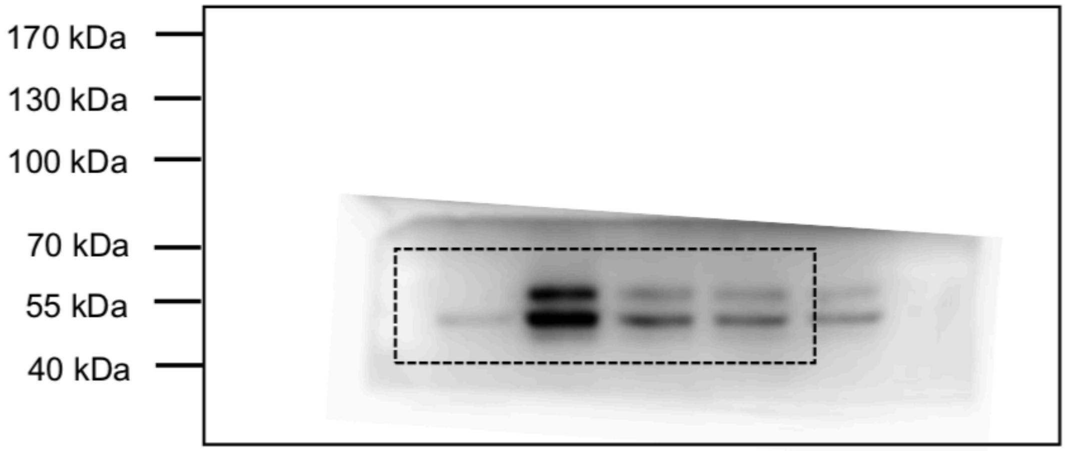

Anti-PPARγ (53,57kDa)

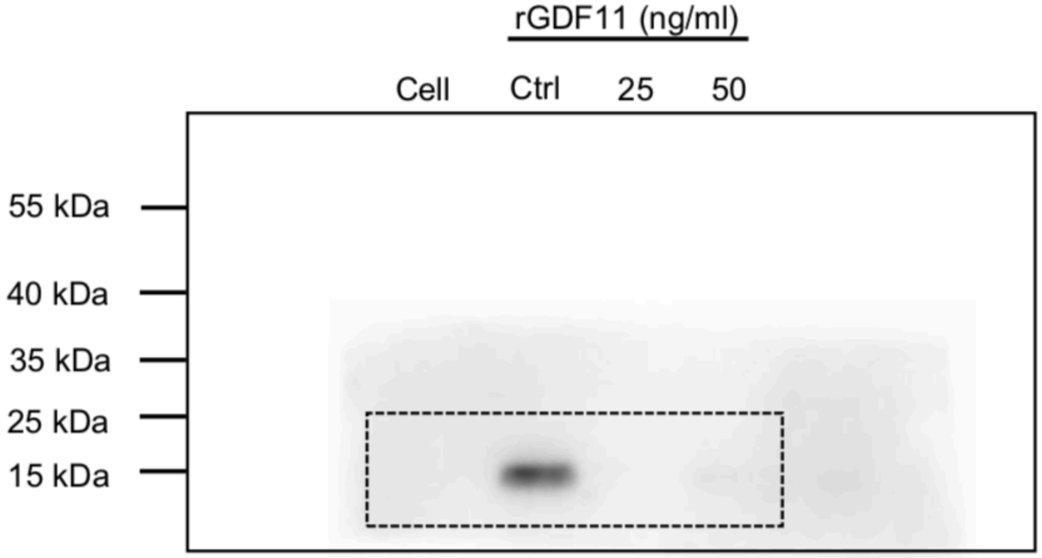

Anti-FABP4 (15kDa)

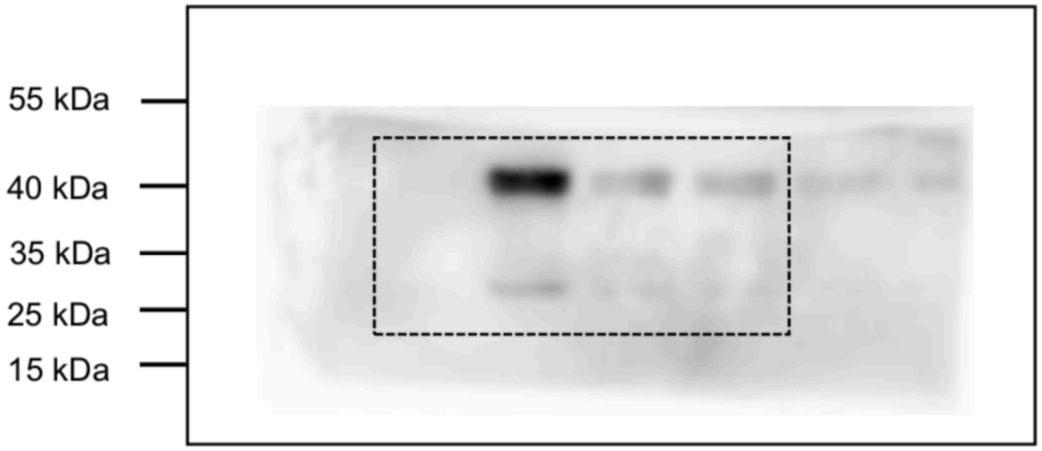

Anti-CEBPα (42,28kDa)

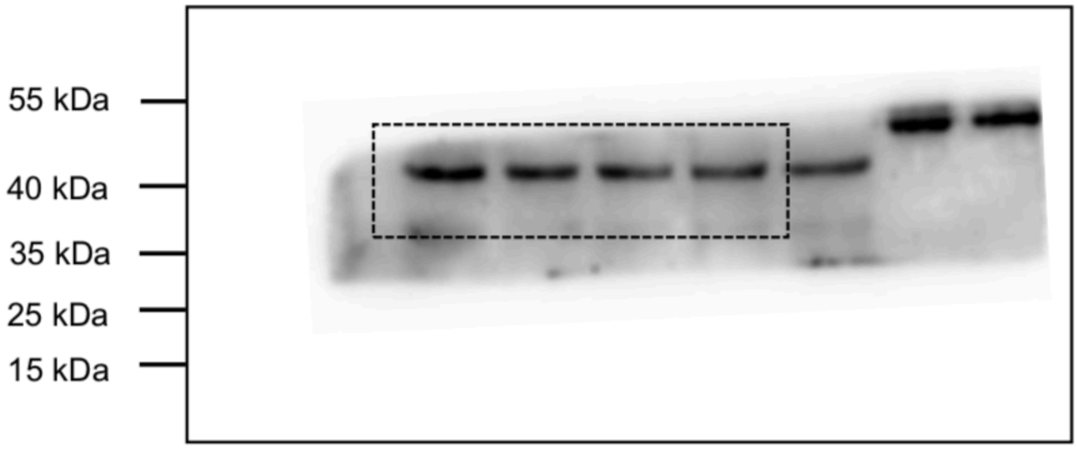

Anti-β-Actin (42kDa)

Uncropped images for Figure 3C

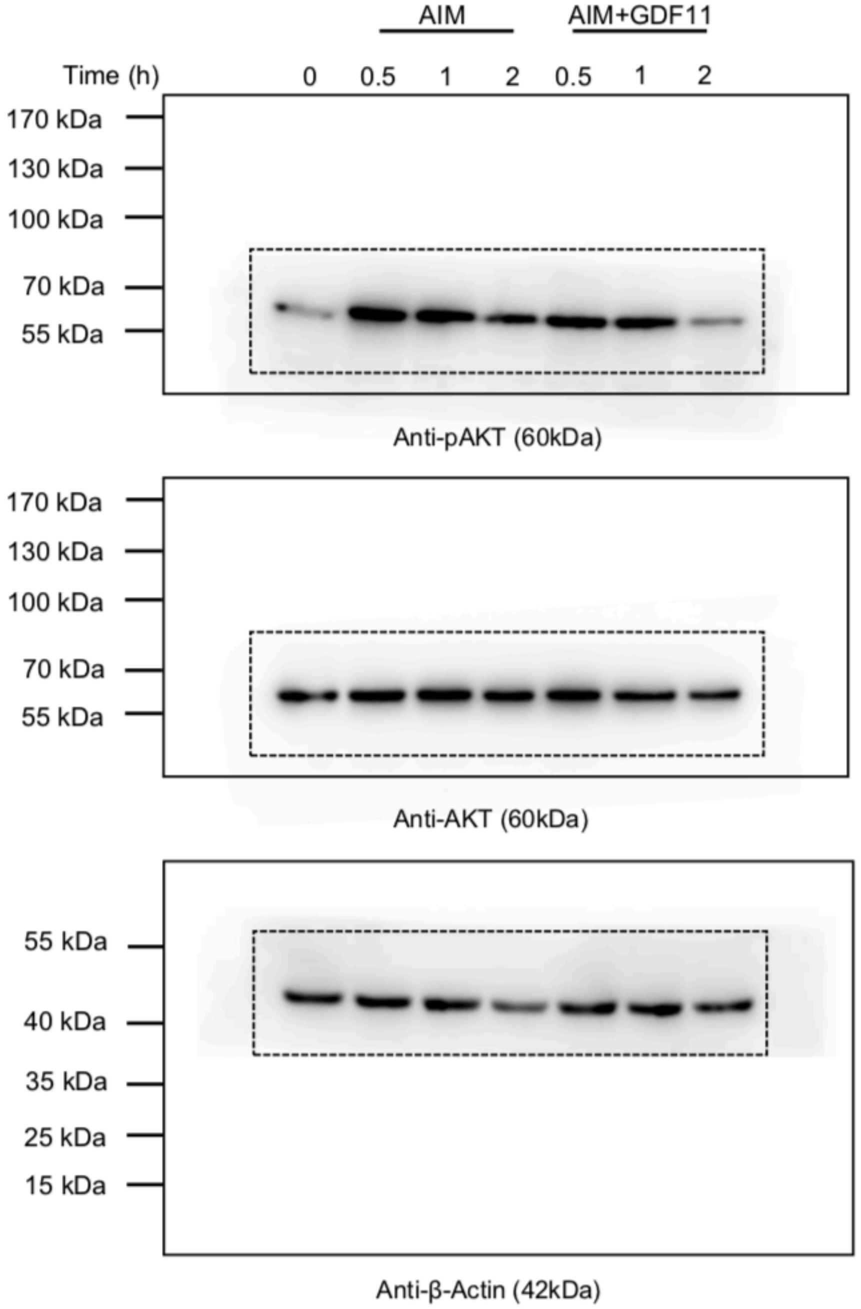

Uncropped images for Figure 4A

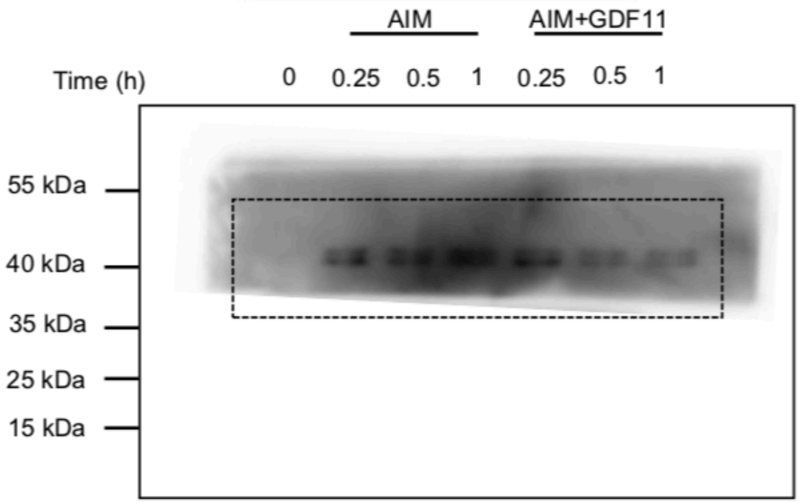

Anti-pERK1/2 (42,44kDa)

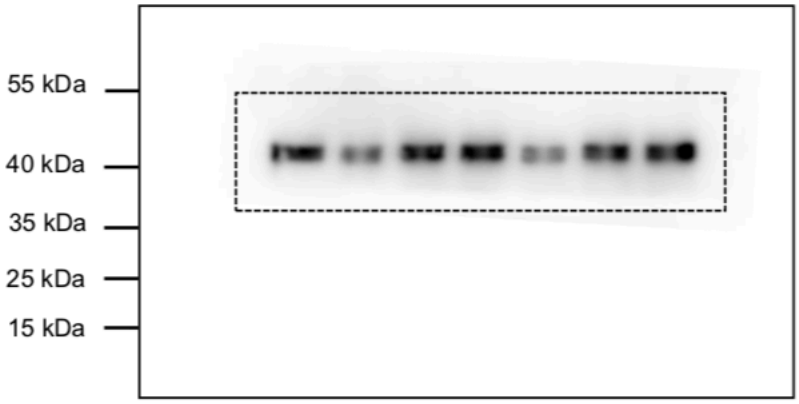

Anti-ERK1/2 (42,44kDa)

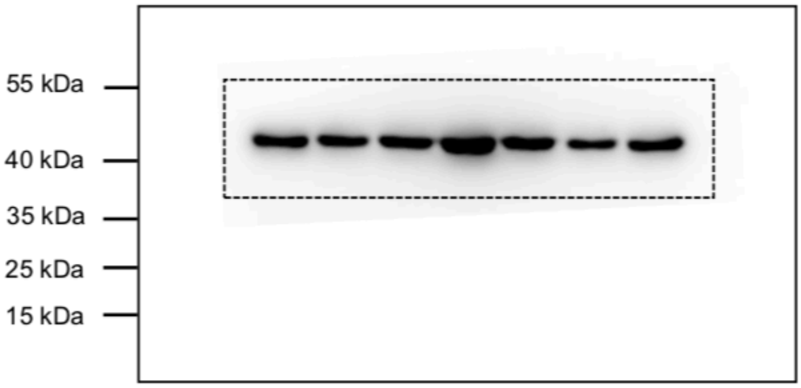

Anti-β-Actin (42kDa)

Uncropped images for Figure 4C

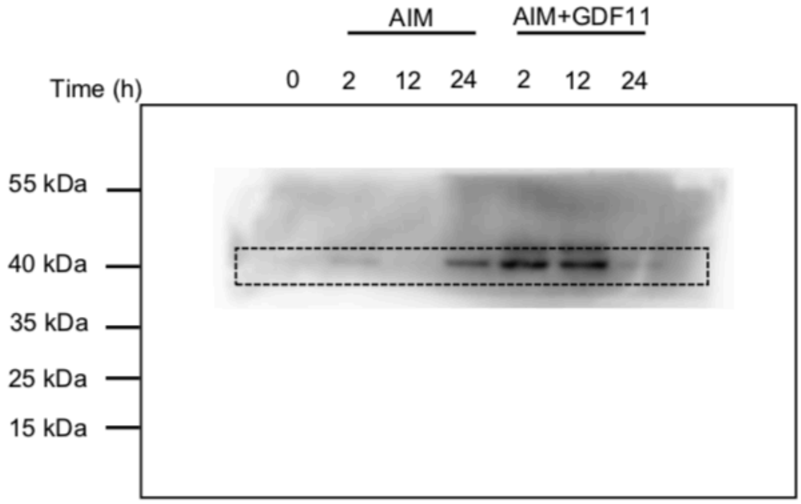

Anti-pP38 (43kDa)

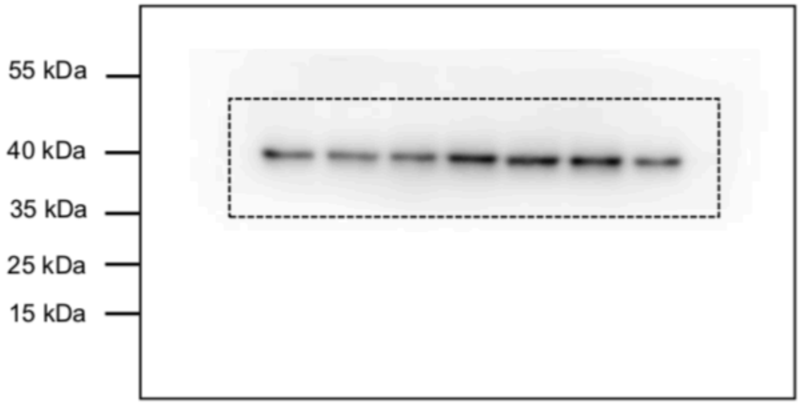

Anti-P38 (40kDa)

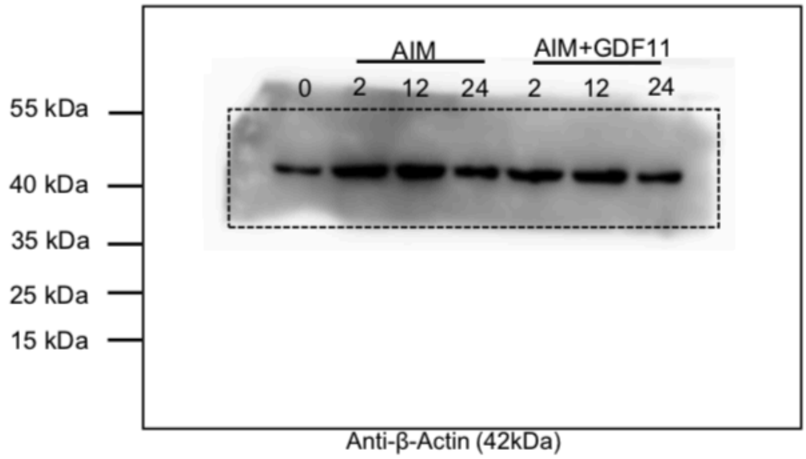

Anti-β-Actin (42kDa)

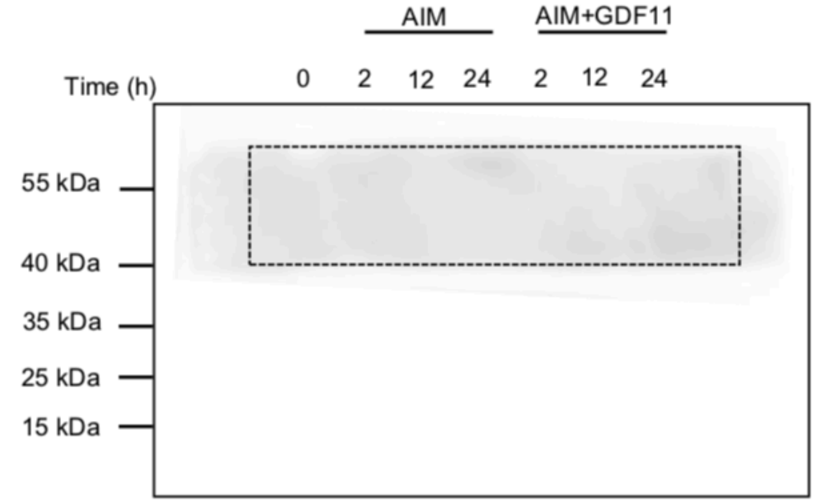

Anti-pJNK (46,54kDa)

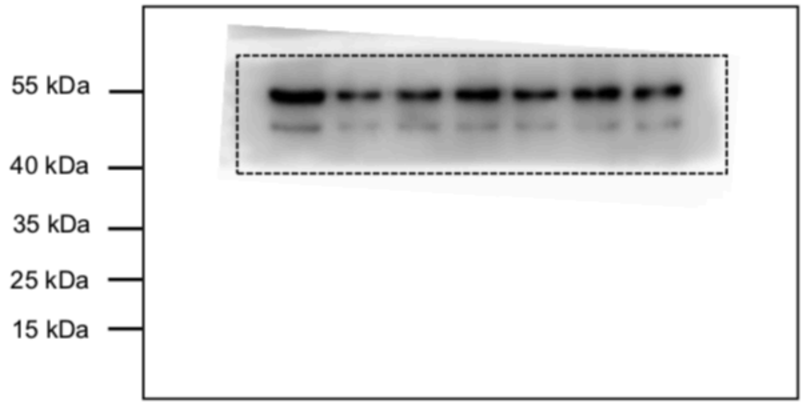

Anti-JNK (46,54kDa)

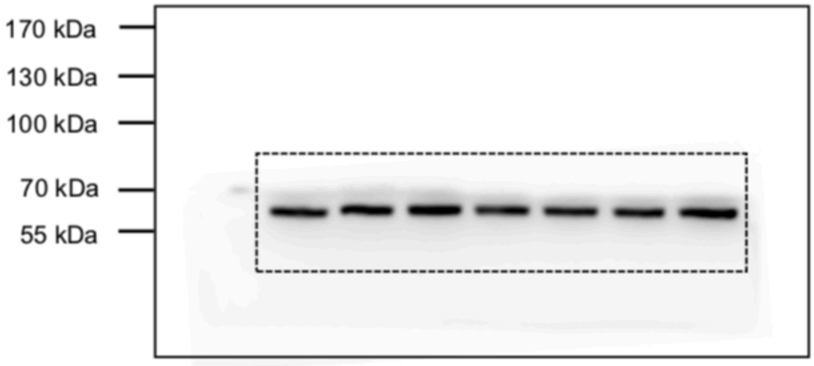

Anti-pSMAD1/5 (60kDa)

Uncropped images for Figure 5A

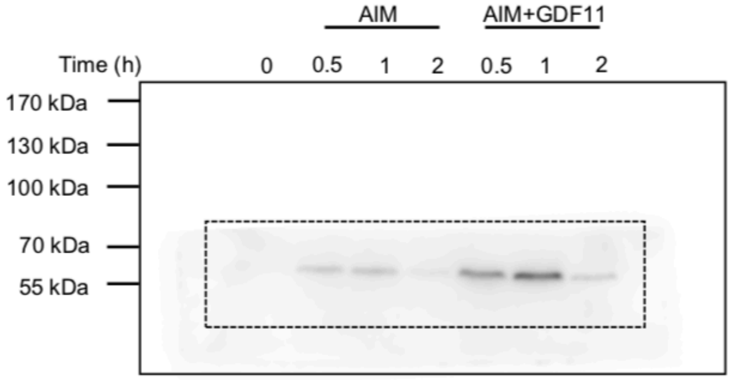

Anti-pSMAD2 (60kDa)

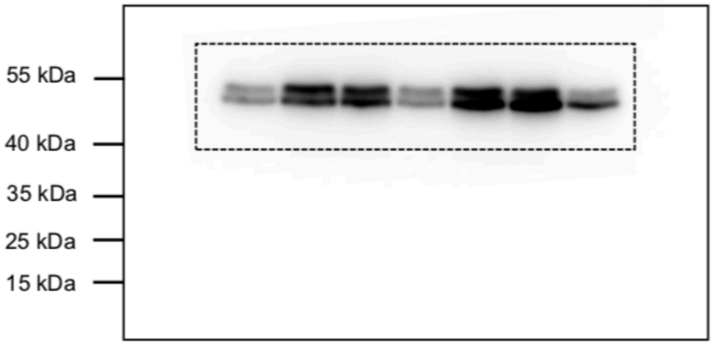

Anti-pSMAD3 (52kDa)

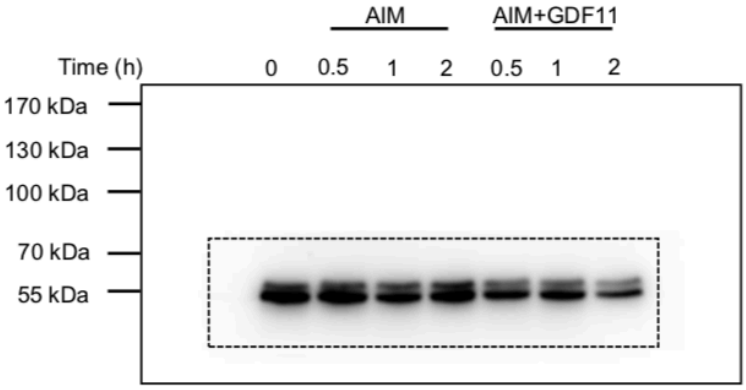

Anti-SMAD23 (52,60kDa)

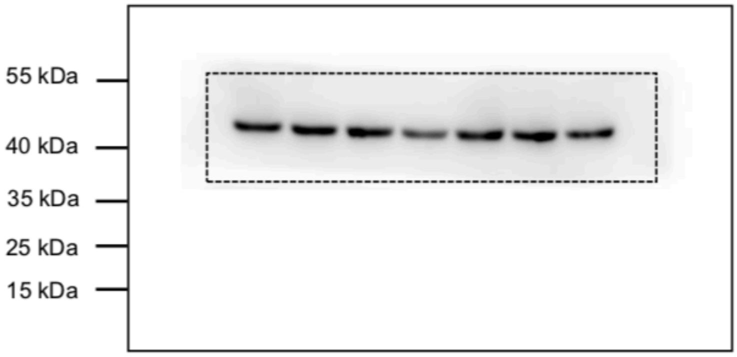

Anti-β-Actin (42kDa)

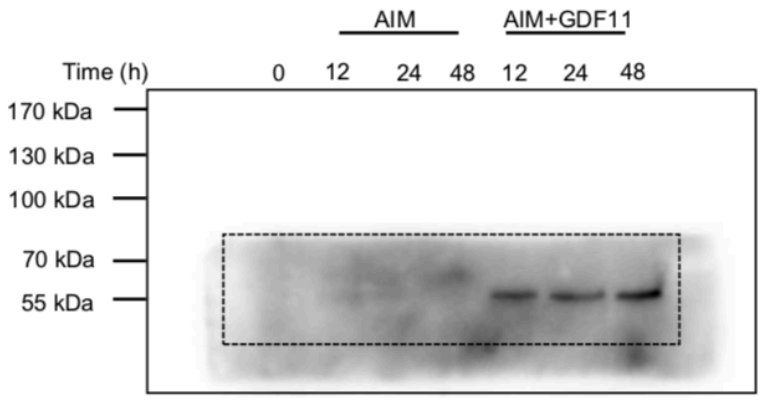

Anti-pSMAD2 (60kDa)

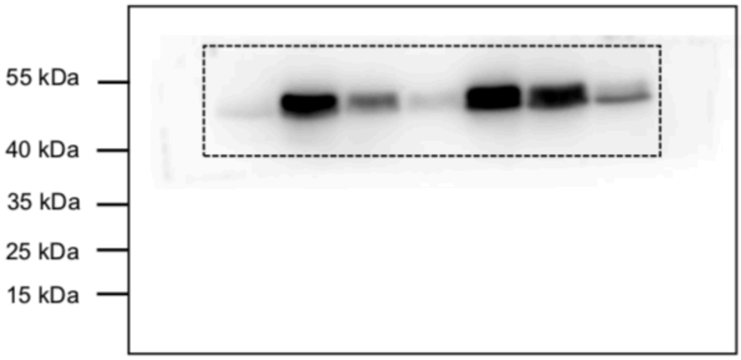

Anti-pSMAD3 (52kDa)

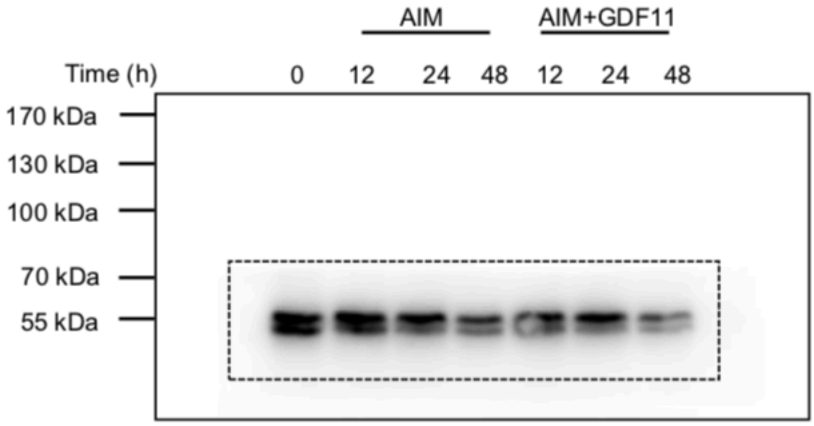

Anti-SMAD23 (52,60kDa)

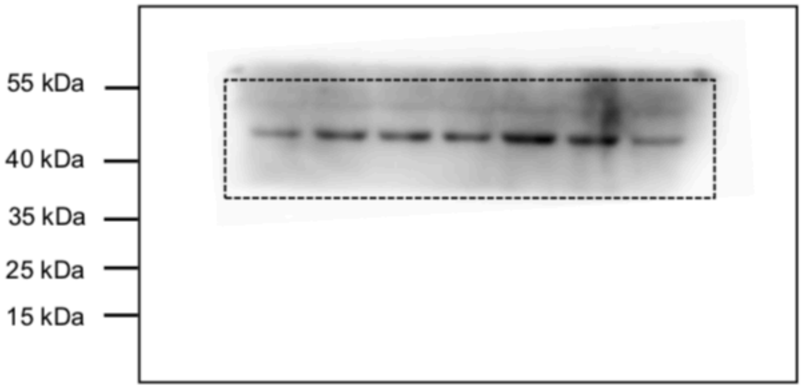

Anti-β-Actin (42kDa)

Uncropped images for Figure 6C

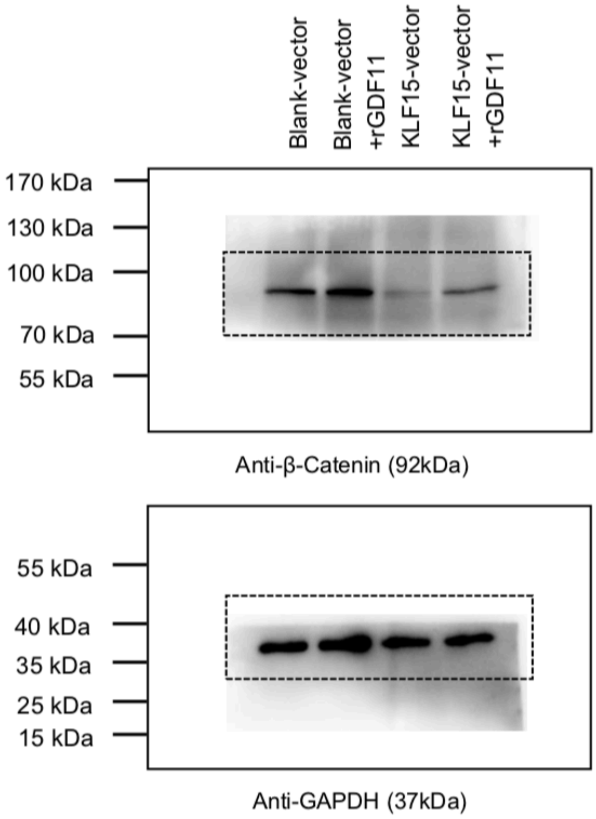

Uncropped images for Figure 6E

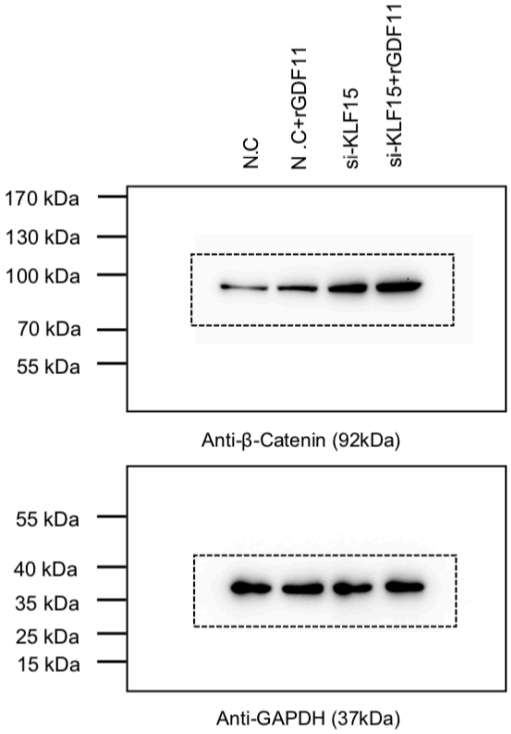

Supplement: Multimedia component 1 [file mmc1.pdf]
